# Chronic Exposure to Youthful Circulation Leads to Epigenetic Reprogramming and Lifespan Extension

**DOI:** 10.1093/geroni/igab046.2551

**Published:** 2021-12-17

**Authors:** Bohan Zhang, David Lee, Alexander Tyshkovskiy, Akshay Bareja, Csaba Kerepesi, Steve Horvath, Vadim Gladyshev, James White

**Affiliations:** 1 Brigham and Women's Hospital, Boston, Massachusetts, United States; 2 Duke University School of Medicine, Durham, North Carolina, United States; 3 Moscow State University, Moscow, Moskva, Russia; 4 UCLA, Los Angeles, California, United States

## Abstract

Heterochronic parabiosis is a powerful rejuvenation model in aging research. Due to limitations in the duration of blood sharing and/or physical attachment, it is currently unclear if parabiosis retards the molecular signatures of aging or affects healthspan/lifespan in the mouse. Here, we describe a long-term heterochronic parabiosis model, which appears to slow down the aging process. We observed a “deceleration” of biological age based on molecular aging biomarkers estimated with DNA methylation clock and RNA-seq signature analysis. The slowing of biological aging was accompanied by systemic amelioration of aging phenotypes. Consistent with these findings, we found that aged mice, which underwent heterochronic parabiosis, had an increased healthspan and lifespan. Overall, our study re-introduces a prolonged parabiosis and detachment model as a novel rejuvenation therapy, suggesting that a systemic reset of biological age in old organisms can be achieved through the exposure to young environment.

